# Crucial Ignored Parameters on Nanotoxicology: The Importance of Toxicity Assay Modifications and “Cell Vision”

**DOI:** 10.1371/journal.pone.0029997

**Published:** 2012-01-10

**Authors:** Sophie Laurent, Carmen Burtea, Coralie Thirifays, Urs O. Häfeli, Morteza Mahmoudi

**Affiliations:** 1 Department of General, Organic, and Biomedical Chemistry, Nuclear Magnetic Resonance and Molecular Imaging Laboratory, University of Mons, Mons, Belgium; 2 Faculty of Pharmaceutical Sciences, University of British Columbia, Vancouver, Canada; 3 National Cell Bank, Pasteur Institute of Iran, Tehran, Iran; 4 Nanotechnology Research Center, Faculty of Pharmacy, Tehran University of Medical Sciences, Tehran, Iran; University of California Merced, United States of America

## Abstract

Until now, the results of nanotoxicology research have shown that the interactions between nanoparticles (NPs) and cells are remarkably complex. In order to get a deep understanding of the NP-cell interactions, scientists have focused on the physicochemical effects. However, there are still considerable debates about the regulation of nanomaterials and the reported results are usually in contradictions. Here, we are going to introduce the potential key reasons for these conflicts. In this case, modification of conventional *in vitro* toxicity assays, is one of the crucial ignored matter in nanotoxicological sciences. More specifically, the conventional methods neglect important factors such as the sedimentation of NPs and absorption of proteins and other essential biomolecules onto the surface of NPs. Another ignored matter in nanotoxicological sciences is the effect of cell “vision” (i.e., cell type). In order to show the effects of these ignored subjects, we probed the effect of superparamagnetic iron oxide NPs (SPIONs), with various surface chemistries, on various cell lines. We found thatthe modification of conventional toxicity assays and the consideration of the “cell vision” concept are crucial matters to obtain reliable, and reproducible nanotoxicology data. These new concepts offer a suitable way to obtain a deep understanding on the cell-NP interactions. In addition, by consideration of these ignored factors, the conflict of future toxicological reports would be significantly decreased.

## Introduction

Due to its social and economic impacts, nanotechnology has become a focus of public interest. Nanotechnology has embedded itself into the fabric of daily life and is expected to have further, as yet unknown, technological impacts owing to its wide reaching use in everything from novel building materials to electronics, cosmetics, pharmaceuticals and general medicine [1].

U.S. and European governments are currently promoting studies that examine the impacts of nanotechnology. A key research report titled “Nanoscience and nanotechnologies: opportunities and uncertainties” was published in 2004 by the Royal Society & Royal Academy of Engineering of Britain [2]. The major recommendation of this report was focused on drawing the attention of scientists on the safe, responsible, and suitable development of engineered nanomaterials. More recently, the Swiss Federal Office of Health, together with the Swiss Federal Office of Environment published an action plan titled “Synthetic Nanomaterials” [3]. Their proposed plan gives researchers and industrial users of nanoparticles (NPs) the means by which to assess their potential risks.

Such hazard assessments are necessary because the novel characteristics of NPs that make them useful in pharmaceutical applications, especially in targeted drug delivery, biomedical imaging, and biosensing, can carry unknown risks [4]. A detailed understanding of the interaction of NPs with living cells, proteins, hormones, or immune factors is thus fundamental to their long-term clinical and commercial viability. It is important to understand how NPs react following biodegradation within the body and whether NPs (or their by-products) are subject to bioaccumulation within cells or organs, thus inducing intracellular changes or inflammatory responses. Although numerous toxicity studies have been performed with NPs, to date these studies have not resulted in the creation of a set of rules applicable to many of the new NPs under development for biomedical applications. They have provided data only on particles of a few specific sizes and with a few defined surfaces. There are great interests in nanosafety issue in prestigious scientific community; for instance, very recently Nature Nanotechnology published a theme issue discussing the importance of nanotoxicology for prioritizing safety studies. We congratulate the journal for opening up this important subject for discussion. With this paper, however, we would like to add two points not mentioned in the Editorial, namely the inadequacy of conventional toxicity assays for the evaluation of NPs and the concept of “cell vision” [5]. We believe these points are crucial for the interpretation, replication, and comparison of nanotoxicology studies and should be added to the detailed characterization of nanomaterials.

## Results and Discussion

### Characterization of SPIONs

**Figure 1:**
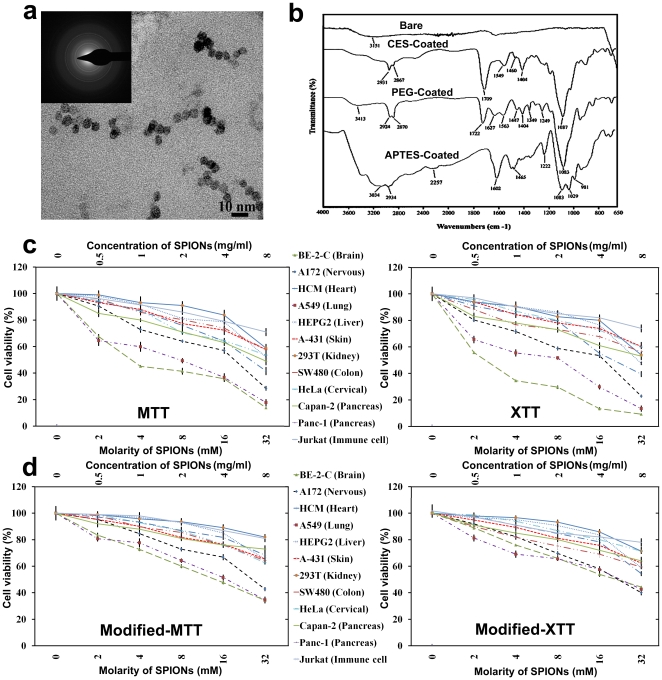
(a) TEM image of monodisperse iron oxide nanocrystals; Inset at the top left illustrates the selected area diffraction pattern of the SPIONs. (b) FTIR spectra of bare and coated-SPIONs with various polymers; and cell viabilities of the conventional (c) MTT- and XTT-assay and (d) modified MTT- and XTT-methods after treatment with various concentrations of CES-grafted SPIONs. Differences between obtained cell viabilities confirm the importance of toxicity method modifications of conventional methods for NPs.

Hydrodynamic sizes and zeta potential of bare and coated NPs are presented in Table 1. DLS results in Table 1 and the TEM image of the synthesized SPIONs (see Figure 1a) confirm the formation of uniform NPs without aggregation. The formation of monodisperse NPs is essential for obtaining reliable and reproducible nanotoxicity results [6]. The FTIR spectrum of bare SPIONs (Figure 1b) exhibited strong bands in the low frequency region (750–400 cm^−1^) due to the iron oxide skeleton. The broad band at 3400–3500 cm^−1^ indicated the presence of surface hydroxyl groups. In the FTIR spectrum of carboxyethylsilanetriol (CES)-grafted SPIONs (Figure 1b), a strong peak at 1709 cm^−1^ is present due to acidic carbonyl (C = O) groups. Absorption bands at 2931 cm^−1^ and 2867 cm^−1^ result from symmetric and asymmetric stretching vibration of methylene groups in CES, respectively. C-O gives a very strong peak at 1087 cm^−1^. The absorption band at 1460 cm^−1^ is due to scissoring bending vibrations of CH_2_ groups. Also the stretching band of O-H groups can be seen around 3000 cm^−1^ as a broad band. By comparing the IR spectra of PEGylated SPIONs (Figure 1b) with CES-grafted SPIONs, successful covalent coupling of CES with PEG is confirmed. In the IR spectrum of PEGylated SPIONs, the intensity of C = O absorption band at 1710 cm^−1^ has decreased and a new band at 1627 cm^−1^ appears, which can be attributed to an amide C = O groups. It shows that by the reaction of NH_2_ groups of the PEG molecules with carboxylic acid groups on the NP surfaces, the number of carboxylic acid groups decreases and new C = O amide groups appear. As a result of the long chain of a PEG molecule and the methylene groups in the PEG structure, the intensity of stretching vibration of CH_2_ (2924 and 2870 cm^−1^) and the C-O band at 1083 cm^−1^ increase. In the IR spectrum of aminopropyltriethoxysilane (APTES)-grafted SPIONs, the absorption band around 2934 cm^−1^ belongs to the stretching vibration of CH_2_ groups. Stretching vibration of C-N band has appeared at 1222 cm^−1^ and the band at 1465 cm^−1^ corresponds to the bending vibration of CH_2_ groups. Furthermore, the broad band above 3000 cm^−1^ is due to N-H stretching, and the band at 1602 cm^−1^ belongs to the N-H bending vibration.

**Table 1:** Comparison of the different SPIONs used in this research. Sizes and zeta potentials are presented as mean ± SD (n = 4).

SPIONs	Size (nm)	Zeta potential (mV)
Bare	13.7±2.1	+43.7±1.7
CES-grafted	13.8±2.1	−15.4±0.5
PEGylated	14.9±1.8	−7.71±0.9
APTES-grafted	17.8±2.6	+32.6±0.3

### Modification of *in vitro* methods

Applying conventional toxicity assays directly to NP suspensions instead of to solutions of the test articles, as generally done, will lead to unreliable toxicity data and will not allow for the correlation of *in vitro* and *in vivo* studies. The reason for the conventional assays to not work, for example, is that NPs exert effects on cell medium components and lead to denaturation of proteins and absorbance of cell medium nutrients, which in turn causes growth delay and toxicity. This was shown in a conventional MTT *in vitro* cell viability assay which led to large errors before modifying the method [7], [8]. Using the modified approach (see Methods for details), the biocompatibility of uncoated superparamagnetic NPs was found to increase significantly (about 20% at an iron concentration of 400 mM (see Figures 1c and 1d for details).

It is shown that during the *in vitro* cytotoxicity assessments, gold NPs can sediment, which means that the concentration of NPs on the cell surface may be higher than the initial bulk concentration, and this could lead to increased uptake by cells and may lead toerrors in toxicity results [9]. In order to check this effect on our particles, the hydrodynamic size of NPs in the cell medium was probed over time (see Table 2 for details). The results confirmed that the synthesized SPIONs were not subjected to sedimentations.

**Table 2:** Time course variations of the hydrodynamic size of various NPs (400 µL with concentrations of 2 mM), while interacting with cell medium (1 mL of DMEM+FBS 10%).

SPIONs	Interaction Time (h)	Size (nm)
Bare	0.1	18.7±4.1
Bare	9	18.8±5.2
Bare	18	19.1±4.8
CES	0.1	21.2±2.4
CES	9	20.8±2.7
CES	18	22.2±1.2
PEGylated	0.1	16.4±2.5
PEGylated	9	18.1±1.9
PEGylated	18	17.8±2.9
APTES	0.1	20.2±3.2
APTES	9	18.9.2±2.7
APTES	18	21.3±2.2

### Effect of Cell “Vision”

Regarding nanomaterial evaluation, the second important concept missing from the Nature Nanotechnology Editorial concerns “cell vision”. “Cell vision” is a complementary concept to protein “corona” and refers to the first contact point of the nanomaterial surface with cells. This contact point is the cell's membrane which is defined by its surface proteins and sugars, and the phospholipid composition of its cell membrane, which all together define how the cell “sees” NPs. There are about 200 types of differentiated cells in the human body, all of which contain cellular membranes of significant variability [10]. Foreign objects (e.g., biomolecules, drugs, and NPs) that come in contact with these cells, or are “seen” by them, thus cause a variable cellular response dependent on the cell type. “Cell vision” for example influences the amount of uptake of foreign objects into cells as well as their fate in the intracellular environment, since membrane transport greatly depends on the composition of cellular membranes.

The concept of “cell vision” can be illustrated by looking at asymmetric cell division. It is fairly well understood that asymmetric cell division has been developed as an evolutionary safety mechanism to ensure that potential toxins, such as damaged proteins or foreign substances, are preferentially inherited by one of the daughter cells upon division [11]. More specifically, one of the daughters becomes the sole carrier of the materials which are potentially damaging to the cell, while the other daughter cell lives on and maintains the health of the wider cell population [12]. A toxicity evaluation which is designed purely on the basis of cell proliferation will not be able to find this effect and cell division asymmetry, which is highly cell type dependent, might introduce significant errors into the interpretation of the results. Modified toxicity assays are thus needed that include the determination of asymmetry. Another effect that might interfere with toxicity assays and should be taken into account in *in vitro* assays include sedimentation, a common occurrence for most NPs. The concentration of NPs on the cell surface becomes thus higher than the initial bulk concentration, which leads to increased cell uptake. Cho *et al.* [9] employed upright and inverted cell culture configurations to show that cellular uptake of gold NPs depends on their sedimentation and diffusion velocities and is independent of size, shape, density, surface coating and initial concentration of the NPs.

Because the concept of “cell vision” is not yet well described, we performed an experiment to show its effect on NP cell uptake and toxicity. Specifically, we probed the impact of superparamagnetic iron oxide NPs (SPIONs) with a narrow size distribution on various human cell lines. Figure 1 shows the uptake of SPIONs and their corresponding toxicity profile to be strongly dependent on cell type. More specifically, the same concentration of SPIONs which caused significant toxicity on the brain–derived neuronal and glial cells and lung cells resulted in very little toxicity on the other cell types. These effects became evident at a concentration of 2 mM (i.e., 114 µg/mL) for neuronal and lung cells, the cell viability of glial cells being diminished to less than 80% at a particle concentration of 4 mM. The highest tested SPION concentration of 32 mM was significantly toxic for the majority of the cell lines. Thus, what the cell “sees”, when it is faced with NPs, is most likely dependent on the cell type.

Using modified MTT and XTT methods, the cell toxicity effects of the SPIONs were significantly reduced. More specifically, the part of cell death which occurred due to changes in cell medium nutrients, was removed and led to more reliable and reproducible toxicity results.

**Figure 2:**
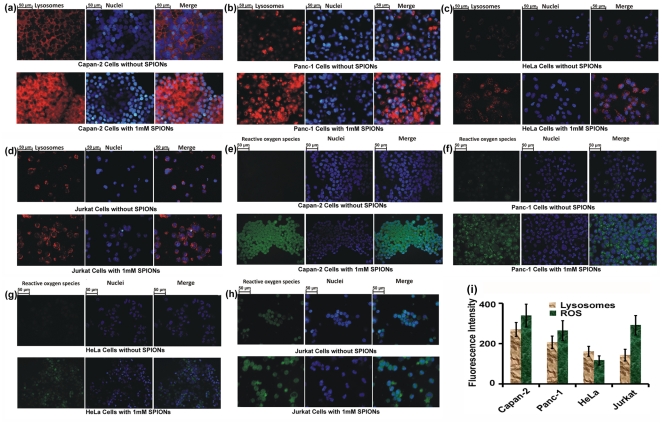
Induced lysosomes in (a) Capan-2, (b) Panc-1, (c) HeLa, and (d) Jurkat cells were obtained upon interaction with CES-coated SPIONs. In live lysosomes assay, the lysosomes and nuclei are seen as red and blue fluorescence, respectively. Induced ROS level in (e) Capan-2, (f) Panc-1, (g) HeLa, and (h) Jurkat cells were obtained upon interaction with SPIONs. In intracellular ROS assay, the ROS level and nuclei are seen as green and blue fluorescence, respectively; (i) fluorescence intensities of induced lysosomes and ROS for all cell lines.

After cellular uptake, SPIONs commonly reside in endosomes or lysosomes where they decompose into free iron, which is slowly released to the cytoplasm and eventually contributes to the total cellular iron pool. The subsequent fate of the iron and its involvement in cell viability and physiology is very complex, and ranges from the stimulation of cell proliferation to variations in the ferritin expression and radical oxygen species (ROS) production. In order to visualize the lysosome induction subsequent to SPION uptake by various cell types, a lysosome tracking assay was employed on living cells and analyzed by fluorescent microscopy. Figures 2a–2d show the varying content of lysosomes after interaction with the same amount of CES coated SPIONs. Under baseline conditions, the lysosomes were well represented in the Capan-2, Jurkat, Panc-1, and HeLa cells with the best representation in the Capan-2 cells. After incubation with SPIONs, additional lysosome formation was strongly induced, although this phenomenon was variable among the investigated cell lines (i.e., Capan-2 (271%)>Panc-1 (207%)>HeLa (163%)>Jurkat (144%)).

Since ROS formation is a frequent consequence of intracellular SPION processing, confocal microscopy analysis was also applied to evaluate the ROS level of the selected cells (see Figures 2e–2h). ROS are produced inside the acidic environment of lysosomes through the reaction of free iron in the form of ferrous ions (Fe^2+^) with hydrogen peroxide and result in the generation of hydroxyl radicals (Fenton reaction) [13]. After intracellular release, the free iron can cross the nuclear or mitochondrial membrane. The hydrogen peroxide and oxygen produced inside mitochondria also undergoes the Fenton reaction, generating hydroxyl radicals and ferric ions (Fe^3+^). The hydroxyl radicals might then indirectly damage DNA, proteins and lipids [14]. Interestingly, various cell types show different ROS levels depending on the pathways they use to defend themselves against foreign substances (i.e., SPIONs).

For most of the cells (see Figure 2i), ROS production appeared to be directly related to the quantity of lysosomes induced by SPION exposure (coefficient of correlation 0.958; see Figure 2 i), where Capan-2 cells showed the highest ROS generation (340%), followed by Panc-1 (265%) and HeLa cells (118%). The endocrine origin of Capan-2 cells and the associated intense metabolism may explain this prominent SPIONs uptake (suggested by the induced lysosomes) and the subsequent ROS production in this cell type. However, this correlation between ROS and lysosome content could not be observed in Jurkat cells, where the ROS generation was superior to that in Panc-1 cells (292%). This could be related to the fact that oxidative stress plays an important role in the regulation of the immune system by a precise control of the lymphocytes' (Jurkat cells) survival [15]. Therefore, the ROS production may be a more intense phenomenon in these cells, which must react to death or survival stimuli in a very well controlled manner.

Cellular particle uptake and ROS confirmed the significant importance of “cell vision” in the interpretation of cytotoxicity data. More specifically, for the achievement of reliable and reproducible toxicity data, it is essential to define the dose and concentration of NPs per cell. As seen from the “cell vision” results (i.e., Figure 2), the SPION concentration per cell was strongly dependent on the cell type after applying the identical SPION amount. Thus, “cell vision” must be considered not only in interpretation of the toxicity data, but also in extension of the obtained data to other cell types.

**Figure 3:**
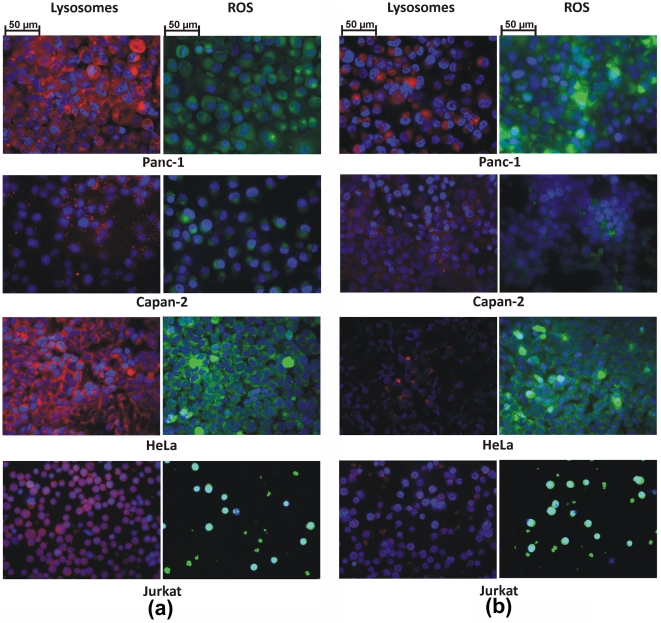
Induced lysosomes and ROS level in various cell lines upon interaction with (a) PEG- and (b) APTES-coated SPIONs.

**Figure 4:**
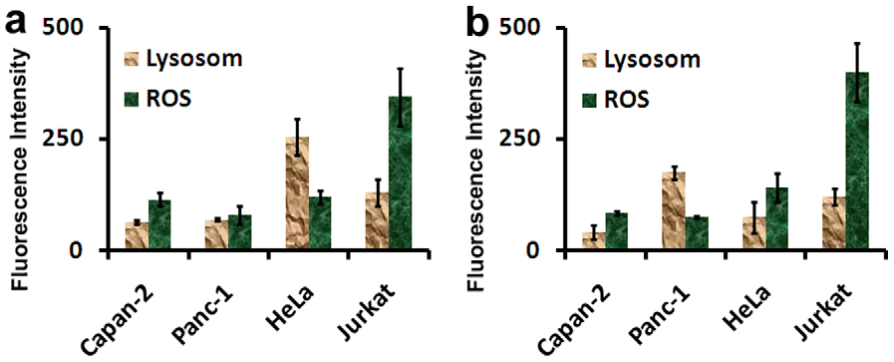
(a) and (b) fluorescence intensities of induced lysosomes and ROS for all cell lines after treatment with PEG- and APTES-coated SPIONs.

In order to probe the effect of particle charge on the “cell vision” idea, neutral NPs (i.e., polyethylene glycol (PEG)-coated SPIONs) and positively charged NPs (i.e., aminopropyltriethoxysilane (APTES)-coated SPIONs) were also synthesized and their lysosome induction and ROS production potential were probed and analyzed by fluorescent microscopy. The results are shown in Figures 3 and 4, and confirmed that the concept of “cell vision” should be considered for all NPs regardless of their physicochemical properties (see Table 3). It is notable that the composition of nanomaterials is recognized as one of the crucial factors that can make significant differences in the composition of surface-associated protein corona [16]. The variation of the protein corona composition can define the amount and fate of particles inside the cells, which strongly affect the toxicity behavior of particles [6].

**Table 3:** Description of the cell lines used in MTT and XTT studies (DMEM: Dulbecco's modified Eagle's medium; Ham's: Nutrient Mixture F-10; FBS: fetal bovine serum; RPMI-1640 (Roswell Park Memorial Institute)).

Cell Code	Cell Type	Culture Medium
BE(2)-C	Human neuroblastoma	1∶1 (DMEM+Ham's F12)+FBS10%
A172	Human glioblastoma	DMEM+FBS10%
HCM	Human cardiac myocytes	1∶1 (DMEM+Ham's F12)+FBS10% supplemented with 5 µg/ml Insulin & 50 ng/ml bFGF
A549	Human lung adenocarcinoma	DMEM+FBS 10%
Hep G2	Human hepatocellular carcinoma	RPMI 1640+FBS 10%
A-431	Human epithelial carcinoma	DMEM+FBS 10%
293T	Human embryonic kidney	RPMI 1640+FBS 10%
SW480	Human colon adenocarcinoma	DMEM+FBS 10%
HeLa	Human cervical adenocarcinoma	MEM+FBS 10%
Capan-2	Human pancreas adenocarcinoma	RPMI+FBS 10%
Panc-1	Human pancreatic carcinoma	DMEM+FBS 10%
Jurkat	Human T cell lymphoblast-like	RPMI+FBS 10%
L929	Mouse connective tissue fibroblast	RPMI+FBS 10%

In summary, we claim that the scientific community cannot assure the readers of the quality of toxicology studies until three major areas have been looked at: (i) the characterization of the nanomaterials to be tested; (ii) the validity and suitability of the selected toxicity methods; and (iii) the influence of “cell vision”. Therefore, we would humbly encourage researchers to not only include modifications in various toxicity protocols to obtain reliable toxicity results of NPs, but to also consider the effect of “cell vision” in the interpretation of their data. As much as possible, these investigations should include looking into interaction and toxicology pathways using newly known cell and molecular biology reactions. New information that arises from such efforts could provide valuable insights into the methods by which to tackle the currently unreliable and difficult-to-reproduce toxicity results. Ideally, these efforts would lead to an improved interpretation and generalization of the scientific results from the nano-toxicology community.

## Materials and Methods

### Synthesis of SPIONs

In order to have NPs with very narrow-size distribution, thermal decomposition was used according to the procedure described before [17]. Briefly, the procedure consists of the preparation of an iron-oleate complex followed by the synthesis of iron oxide nanocrystals. For the preparation of the iron-oleate complex, 10.8 g of iron chloride (FeCl_3_·6H_2_O, 40 mmol, 98%, Sigma-Aldrich, Munich Germany) and 36.5 g of sodium oleate (120 mmol, TCI, Sigma-Aldrich, 95%) was transferred and well-dissolved in a solvent blend of 80 ml ethanol, 60 ml distilled water and 140 ml hexane. The obtained solution was heated to and kept at 70°C for 1 hour, followed by washing of the upper organic layer, which contains the iron-oleate complex, several times with distilled water in a separatory funnel. Hexane was then evaporated off, resulting in the iron–oleate complex in waxy solid form. The prepared iron-oleate complex (36 g) was mixed with 5.7 g of oleic acid (20 mmol, 90%, Sigma-Aldrich) and was dissolved in 200 g of 1-octadecene (90%, Sigma-Aldrich) at 30°C. The resulting solution was heated to 320°C at a constant heating rate of 5°C per minute. After 30 min incubation, the resulting SPIONs suspension was cooled to room temperature, and 500 ml of ethanol added to precipitate the SPIONs. They were separated by a strong magnetic field gradient from a permanent cylindrical NdFeB magnet (4×3×3 cm^3^), and interacted with dimethyl sulfoxide for 10 hrs with CES polymer following by washing several times with 1 M HNO_3_.

### Preparation of SPIONs with CES

Carboxyethylsilanetriol was used as coating. Briefly, 100 mL of SPIONs (300 mM iron) was added to 100 mL of DMF. Then, 45 mL of 0.15 M CES was slowly added before adding 25 mL of water followed by 15 mL of 1 M NaOH at room temperature and under homogenization (about 8,000–24,000 rpm). The suspension was heated to 100°C for 24 h under continuous stirring, the SPIONs precipitated by addition of a mixture of acetone/ether (50/50) and magnetically collected. The precipitate was washed with acetone several times and finally dispersed in water. An excess of the silane derivative and other chemicals were removed by dialysis using a dialysis bag (Spectrum Laboratories, Inc; 10,000 Da MWCO) for 48 h in water. The obtained CES ferrofluids ( = SPIONs) were kept at 4°C for future usage.

### Grafting of SPIONs with APTES

Fifteen mL of APTES was dissolved in 50 mL methanol and drop-wise added to a suspension of SPIONs (20 mL, [Fe] = 0.3 M). After stirring for 24 h at room temperature, 20 mL of glycerol was added to the mixture and subsequently methanol and water were removed by rotary evaporation. Next, 50 mL of acetone was added and after mixing the SPIONs were separated by magnetic decantation. This was repeated several times and the SPIONs were finally dispersed in 40 mL of water and dialyzed for 48 h (MWCO = 10,000).

### PEGylation of SPIONs

PEGylation of SPIONs was done by an amidation reaction between amino-polyethylene glycol and carboxylic acid groups on the surface of CES-modified SPIONs. Briefly, 0.156 g of N-(3-dimethylaminopropyl)-N′-ethylcarbodiimide hydrochloride, as a carboxyl activator, and 0.254 g of PEG-NH_2_ (Sigma-Aldrich) were added to 2 mL of SPIONs-CES (2 mg/mL) and the mixture was stirred at room temperature for 24 h and washed several times by ultrafiltration on a 30 kD MWCO membrane.

### Characterization of SPIONs by particle size distribution, zeta potential and transmission electron microscopy

Transmission electron micrographs (TEM) of the SPIONs were deposited on carbon coated copper grids and analyzed by TEM operating at 200 kV. The hydrodynamic diameters and zeta potentials of the SPIONs in water were measured using a Malvern Zeta Sizer Nano S-90 dynamic light scattering (DLS) instrument.

### Fourier Transform Infrared (FTIR) Spectroscopy

FTIR spectra were obtained using a PerkinElmer Spectrum 100 spectrometer in the range of 4000–650 cm^−1^, and each spectrum was obtained by averaging 32 interferograms with a resolution of 4 cm^−1^. Samples for FTIR analysis were prepared by lyophilizing SPION suspensions in water and a thin film of lyophilized SPIONs was placed on the attenuated total reflectance crystal for spectral recording.

### Cell Culture and Treatments

Cell lines from different origins (e.g., brain, heart, lung, liver, skin, kidney, colon, and cervix) were used for the cytotoxicity assays (see Table 3). Human HCM (Heart), BE-2-C (Brain) and 293T (Kidney) cell lines were obtained from the Riken Cell Bank (Tsukuba, Japan) and were cultured in Eagle's minimal essential medium (MEM) (Nissui, Tokyo, Japan) supplemented with 1% non-essential amino acid (Invitrogen, Carlsbad, CA), 10% fetal bovine serum, and 60 mg/mL kanamycin at 37°C and 5% CO_2_. Panc-1 and Capan-2 cells were kindly provided by Dr. Daizy Flamez (Free University of Brussels, Experimental Medicine Laboratory, Belgium). Panc-1 cells were cultured in pyruvate-free DMEM culture medium (Lonza, Verviers, Belgium) supplemented with 10% fetal bovine serum, non-essential amino acids (both from Invitrogen), and penicillin/streptomycin (Lonza). Capan-2 cells were cultured in advanced RPMI-1640 culture medium supplemented with 10% fetal bovine serum, glutamax (all from Invitrogen), and penicillin/streptomycin (Lonza). Jurkat cells (gift from Prof. Leo Oberdan, Free University of Brussels, IBMM, Belgium) were cultured in RPMI-1640 (Sigma-Aldrich, Bornem, Belgium) at a concentration of less than 1×10^6^ cells/mL supplemented with 10% newborn calf serum, antibiotic-antimycotic (both from Invitrogen) and heat inactivated. HeLa cells were cultured in MEM culture medium supplemented with 10% fetal calf serum, glutamax, antibiotic-antimycotic, non-essential amino acids, and sodium pyruvate (all from Invitrogen). Other cells were obtained from the National Cell Bank of Iran (NCBI) and Pasteur Institute of Iran and grown with specific media (Table 3).

### MTT and XTT assays

All cell lines were seeded into a 96 well-plate at a density of 10,000 cells (2,500 cells for HCM cells) per well in 100 µL of medium. After 24 h, 40 µL of the corresponding medium containing various concentrations of SPIONs (2–32 mM) was added to each well. Forty microliter of base medium for each cell line was added to negative control wells.

Cytotoxicity was assessed using the MTT (3-(4,5-dimethylthiazol-2-yl)-2,5-diphenyltetrazolium bromide) and XTT (sodium(2,3-bis(2-methoxy-4-nitro-5-sulphophenyl)-2H-tetrazolium-5-carboxanilide) assays 24 h after the incubation with SPIONs, 100 µL of MTT (0.5 mg/mL) was added to each well. Following incubation, the medium was removed and formazan crystals were solubilized by incubation for 20 min in 150 µL of isopropanol. The absorbance of each well, which assesses viable cells, was read at 545 nm on a microplate reader (Stat Fax-2100, AWARENESS, Palm City, USA). Regarding XTT assay, 24 h after the incubation with SPIONs, 50 µL of XTT labeling mixture was added to each well and incubated for 18 h, after which the amount of formazan crystals were measured using a plate reader. For the MTT and XTT studies, all experiments were carried out in triplicate (i.e., three 96 plates; total 15 repeats). The results were statistically processed for outlier detection using a “T procedure” [18] in the MINITAB software (Minitab Inc., State College, PA). Statistical differentiations were made by one-way analyses of variance (ANOVA), for which p<0.05 was considered as statistically significant.

### Assay of reactive oxygen species (ROS)

For visualization by confocal microscope, the adherent cells (Panc-1, Capan-2, and HeLa) were seeded on cover slips before incubating with various compounds, while Jurkat cells were incubated in suspension. The cells were incubated for 24 h with SPIONs (55.845 µg iron/mL = 1 mM of iron) that were added to the culture medium. Control cells were grown without SPIONs. The cells were then washed three times with ice-cold PBS and incubated for 1 h with 10 mM 5-(and-6)-chloromethyl-2,7-dichlorodihydrofluorescein diacetate acetyl ester (H_2_DCFDA, Invitrogen, Merelbeke, Belgium) in PBS at 37°C. The cells were subsequently washed three times with ice-cold PBS, fixed with 2% paraformaldehyde for 15 min at room temperature and the cell-coated cover slips were finally mounted on microscope slides by using Vectashield mounting medium with DAPI (Vector Labconsult, Brussels, Belgium) [19]. The method of ROS labeling was slightly modified for Jurkat suspension cells. The cells (2×10^6^/ml) were incubated (45 min, 37°C) with 25 µM H_2_DCFDA in HBSS. Five minutes before ending the incubation with H_2_DCFDA, a solution of Hoechst 33342 dye was added at a final concentration of 1 µM. The cells were then rinsed three times with HBSS, the supernatant being removed by centrifugation. At the end, the cells were mounted on microscope slides after resuspending them in 25 µl HBSS. All the samples were observed on a confocal microscope (Leica Microsystems, Groot Bijgaarden, Belgium). A semi-quantitative analysis of the microscope pictures has been performed by using the ImageJ image analysis software, the fluorescence intensities being related to the cell number per picture. The results were expressed as percentage of cell labeling in SPION-treated samples as compared to control cells.

### Lysosome labeling

Panc-1, Capan-2, Jurkat, and HeLa cells were labeled with Image-iT™ LIVE lysosomal and nuclear labeling kit (Molecular Probes, Invitrogen), which provides a red-fluorescent LysoTracker®Red DND-99 dye for lysosome staining, and a blue-fluorescent Hoechst 33342 dye for staining the nucleus. The adherent cells (Panc-1, Capan-2, and HeLa) were seeded on cover slips before incubating with various compounds. Jurkat cells, which are not adherent, were incubated in suspension, the various compounds being removed by centrifugation. The cells were incubated (37°C, 24 h) with SPIONs that were added in the culture medium at a concentration of 55.845 µg/ml (1 mM of iron). Control cells were left not incubated with SPIONs. After rinsing the cells with Hanks Buffered Salt Solution (HBSS), they were labeled with Image-iT™ LIVE lysosomal and nuclear labeling kit according to the supplier's instructions. Briefly, the cells were incubated for 5 min with 2 µg/ml of Hoechst 33342 solution, followed by 1 min incubation with 100 nM of LysoTracker Red DND-99®. The cells were rinsed two times with HBSS after each dye. The living cells were finally mounted in HBSS on microscope slides and observed on a DM2000 Leica microscope (Leica Microsystems, Groot Bijgaarden, Belgium), the pictures being acquired with a Leica DFC 290 camera. The microscope pictures were finally analyzed by using the ImageJ software as described above.

### Protocol for Modification of MTT and XTT Method for Toxicity Evaluation of NPs

The core hypothesis of the protocol is to obtain reliable and reproducible toxicity results by understanding the effect of NPs on the cell medium, in particular the interaction of NPs with biomolecules. It is now well-recognized that biomaterials (e.g., implants and medical devices) are covered by biomolecules (e.g., proteins, natural organic materials, detergents, and enzymes) immediately upon entrance of the biomaterial into a biological medium [16]. Due to their extremely high surface to volume ratio, NPs have a very active surface chemistry in comparison to bulk biomaterials. For this reason, in biological applications, they tend to reduce their large surface energy by interaction with the medium components in which they are dispersed. Thus, dispersing of NPs in a biological medium results in their surfaces (as with bulk materials) being covered by a dynamic layer of biomolecules [16]. As a result, the composition of cell medium which is essential for cell nutrition can be significantly changed, leading to non-optimal medium composition which is not perfect for cell maintenance and causes undesired cell death. This effect was not considered in the conventional *in vitro* examination methods. Here, in order to remove the effect of protein removal from the cell culture, we proposed a modified method as follows [20]:

- Introduce the NPs to the cell medium (without cells).
- Leave the cell medium in contact with NPs for a period of 24 h, for the formation of relatively static hard corona proteins at the surface of SPIONs.
- Remove excess medium using MACS®(magnetic separation) system.
- Redisperse the particles with stable protein corona in fresh medium.
- Apply the surface saturated SPIONs to the cells and perform toxicity assays.

Using this modified method, the cell medium will not encounter significant protein changes and thus avoid errors, which arose from cell culture composition variation in the non-modified method.
